# Impact of Acute Kidney Injury on Critically Ill Children and Neonates

**DOI:** 10.3389/fped.2021.635631

**Published:** 2021-04-26

**Authors:** Bassil Leghrouz, Ahmad Kaddourah

**Affiliations:** ^1^Pediatric Nephrology and Hypertension Division, Sidra Medicine, Doha, Qatar; ^2^Weill Cornel Medical College, Ar-Rayyan, Qatar

**Keywords:** acute kidney injure, volume overload, critically ill children, neonatal intensive care unit, COVID-19

## Abstract

Acute kidney injury (AKI) is a clinical syndrome that manifests as an abrupt impairment of kidney function. AKI is common in critically ill pediatric patients admitted to the pediatric intensive care units. AKI is a deleterious complication in critically ill children as it is associated with increased morbidity and mortality. This review provides an overview of the incidence, morbidity, and mortality of AKI in critically ill children in general and specific cohorts such as post-cardiac surgeries, sepsis, critically ill neonates, and post stem cell transplantation.

## Introduction

Acute kidney injury (AKI) refers to a clinical syndrome manifested as abrupt impairment of kidney function. Although the first systematic terminology and classification of AKI were not developed till 2002 by the Acute Dialysis Quality Initiative (ADQI) group, the clinical manifestations and the deleterious impacts of this syndrome were reported in ancient medical scripts. In his Aphorisms written sometime in 400 *BCE*, Hippocrates described oliguric kidney failure complicating a febrile illness and described the generalized edema as “leucophlegmatia,” which means an overabundance of white phlegm as an attempt to interpret the cause of the white skin color seen in such edematous patients (1). Throughout history, researchers and clinicians gave this syndrome different terms, such as “ischuria renalis” in the 1800s, acute Bright's disease in the early 1900s, and “war nephritis” during the First World War (2, 3). However, the description of this syndrome was not based on a systematic approach till the “Risk, Injury, Failure, Loss, and End-Stage (RIFLE)” criteria were developed by the ADQI working group in 2004 in adults (4). Since then, the RILFE criteria were modified and refined to include more precise criteria and different terminology. In 2007, “pediatric” RIFLE criteria were adopted for children (5) and the acute kidney injury network (AKIN) working group added further criteria and modified the staging of AKI severity (6). Finally, a comprehensive definition and staging that take into consideration the previously described criteria were introduced by the Kidney Disease Improving Global Outcomes (KDIGO) in 2012 (3) as shown in Table 1.

**Table 1:** The kidney disease improving global outcomes (KDIGO) definition and classification of acute kidney injury (AKI)^*^.

Definition		
1. Increase in sCr ≥ 0.3 mg/dl (≥26.5 μmol/L) within 48 h; OR		
2. Increase in sCr ≥ 1.5 times baseline, which is known or presumed to have occurred within the prior 7 days; OR		
3. UOP < 0.5 ml/kg/h for 6 h.		
**AKI severity stages**		
	**Per sCr criteria**	**Per UOP criteria**
Stage 1	1.5–1.9 times baseline OR	
	≥0.3 mg/dl (≥26.5 μmol/L) absolute increase.	<0.5 ml/kg/h for 6–12 h
Stage 2	sCr ≥ 2–2.9 times baseline	UOP < 0.5 ml/kg/h for ≥12 h
Stage 3	sCr ≥ 3 times baseline OR	
	Increase in sCr to ≥4 mg/dl (≥353.6 μmol/L) OR	
	Initiation of kidney replacement therapy OR	
	In patients < 18 years, decrease in eGFR < 35 ml/min/1.73 m^2^	<0.3 ml/kg/h for ≥24 h OR
	Anuria for ≥12h	

* *Adopted and modified from (3)*

*sCr, serum creatinine; UOP, urine output; eGFR, estimated glomerular filtration rate*.

This chapter provides an overview of AKI epidemiology in critically ill pediatric patients and its associated morbidity and mortality. Additionally, we looked into the epidemiology and outcomes of AKI in specific populations of critically ill children.

## Incidence of AKI in Hospitalized Children

AKI is common in hospitalized pediatric patients with variably reported incidences ranging from 0.34% up to 5% in different studies (7, 8). It is more common in critically ill children than other hospitalized children, with a reported incidence of 30–50% (9, 10). This high incidence rate sets AKI as the commonest medical complication in critically ill pediatric patients admitted to pediatric intensive care units (ICU) (7).

AKI rates increased 20-fold, from 0.5 to 9.9 cases per 1,000 hospitalized children between 1982 and 2004 according to a Thai study (11). The increase of the incidence is likely influenced by many factors such as the increased awareness of AKI as a common complication in the ICU population specifically after the evolution of systematic definitions of AKI. Additionally, the increased utilization of nephrotoxic medications (8–10, 12) and the advanced interventional and medical approaches to support critically ill children such as cardiopulmonary bypass (CBP) surgeries, extracorporeal membrane oxygenation (ECMO), solid organ, and stem cell transplantations could contribute to the increased incidence in of AKI.

The pathophysiology of AKI in critically ill patients is multifactorial. It varies in different areas of the world; it can occur secondary to kidney hypo-perfusion (e.g., cardiac dysfunction, hypotension, severe dehydration, bleeding, sepsis, or significant ascites impairing kidney perfusion), kidney tissue injury (secondary to prolonged impaired kidney perfusion or nephrotoxic medications) (10, 13–15), or less commonly due to primary kidney diseases like hemolytic uremic syndrome and glomerulonephritis in <10% of the cases (11, 16).

## Impact of AKI on Pediatric ICU Population

### Impact of AKI on ICU Population Collectively

The AWARE (Association Worldwide AKI Renal Angina and Epidemiology) study, published in 2017, comprehensively delineated AKI in children admitted to ICU. The (AWARE) study (17) is one of the most inclusive published studies in the epidemiology of AKI in critically ill children regardless of the underlying cause of ICU admission. This international observational study involved 32 pediatric ICU centers from the world, mainly from North America. The study enrolled more than 4,600 critically ill children from 3 months up to 25 years old. The study excluded patients with chronic kidney disease who have estimated glomerular filtration rate (GFR) of < 15 ml/min/1.73 m^2^, patients on dialysis, kidney transplant patients admitted within 90 days of transplantation, patients admitted to ICU within 3 months following surgical correction of congenital heart disease, patients with uncorrected congenital heart disease, and patients post-cardiac catheterization. The authors reported AKI incidence using UOP or Cr KDIGO definition as 26.9% (95% CI: 25.6–28.2) during the first 7 days of ICU admission. 11.6% of critically ill children had severe AKI defined as stage II or stage III AKI using serum creatinine and/or urine output KDIGO criteria.

Severe AKI was a statistically independent risk factor for mortality after adjusting for the diagnosis at ICU admission, coexisting medical conditions, the severity of illness scores, and utilization of mechanical ventilation, vasoactive support, ECMO, and renal replacement therapy. The adjusted odds risk of mortality on day 28 of ICU admission was 1.77 in patients with severe AKI. Moreover, severe AKI was independently associated with other secondary outcomes such as increased kidney replacement therapy utilization, increased mechanical ventilation days, and ICU stay length.

In a *post hoc* analysis in the AWARE data (18), the authors highlighted the importance of utilizing both urine output and serum creatinine criteria to identify AKI. They found that isolated use of the creatinine criteria would have missed nearly a third of AKI cases as the creatinine-missed cases met only the KDIGO UOP definition portion. The 28-day mortality rate was similar for those who met stage II or III by only serum creatinine criteria (6.7%) and those who met stage II or III by only urine output criteria. Moreover, the mortality rate was much higher (38%) in those who met both criteria.

The majority of childhood AKI studies did not utilize the urine output criteria. This can be attributed mainly to the fact that the majority of critically ill children do not have good documentation of urine output, as there is a general push back not to insert Foley catheters to prevent urinary tract infections. Even when a urinary catheter is inserted, AKI is usually hard to capture because the urine output is a dynamic measure that requires evaluation over different periods of time for which special programming in the electronic medical record systems is needed to be reliably captured. However, despite the challenges, oliguria remains a crucial component to recognize AKI as the drop in urine output should prompt evaluation of kidney function and more careful management of fluids and nephrotoxic agents.

### Impact of AKI on Children With Congenital Heart Disease Undergoing Cardiac Surgery

AKI following post-cardiac surgery is a known complication in children undergoing cardiac surgery, mainly when utilizing CBP. Indeed, this cohort of critically ill children was the initial focus of childhood AKI research to understand the impact of the biomarkers on AKI. The timed and isolated risk factor of inducing AKI by applying the CBP made this cohort of patients the ideal cohort to study the consecutive pathological changes after initiating the bypass. A significant portion of the data and knowledge regarding the diagnostic utilities, such as the sensitivity and the positive predictive value of the novel tubular injury markers, were acquired by studying these biomarkers in this cohort. Cardiopulmonary bypass is associated with hypotension, impaired kidney perfusion, non-pulsatile perfusion, the release of inflammatory markers, kidney hypoxia, ischemia, and reperfusion injury and tissue damage secondary to oxidative stress; all of these factors contribute to developing AKI post-cardiac surgery (19, 20).

The incidence rate varies from one center to another, but most pediatric studies reported that 30–50% of children and up to 60% of neonates develop AKI after open-heart surgery (21–23). Many well-identified risk factors increase the risk of AKI following cardiac surgeries, such as low weight with the highest risk in the neonates and weight <5 kg, the complexity of the cardiac defect, high pre-operative serum creatinine level, decreased post-operative cardiac output, and high inotropic requirement (19–21, 24–28). Several studies have found out that there is a potential direct association between prolonged CPB times and the development of AKI. In a prospective pediatric multicenter study, the risk of developing AKI was 51 and 70% for bypass duration of more than 120 and 180 min, respectively (19). In a meta-analysis of nine studies, the authors concluded that longer CPB times are strongly associated with a higher incidence of AKI (29). Despite the consensus of the plausibility of the association between AKI and CBP duration, it is worth mentioning that this association was not observed in other studies. Some other technical and medical factors such as the flow rates, temperature, patient's hematocrit, and cardiac index probably play more interactive roles in determining the risk of AKI rather than the CBP time itself (30–32).

Development of AKI in post-cardiac surgery has been reported to be independently associated with increased morbidity and mortality in children, hospital length of stay, prolonged mechanical ventilation duration, more inotropic requirement, and increased mortality rate (17, 19, 20, 33).

### Impact of AKI on Children Receiving ECMO

The Kidney Intervention During Extracorporeal Membrane Oxygenation (KIDMO) study is probably the most comprehensive study devoted to evaluating AKI and kidney supportive measures for critically ill children receiving ECMO (34). The study group utilized data of more than 800 pediatric ECMO patients from multiple centers to describe the incidence and timing of AKI by utilizing serum creatinine KDIGO criteria and to investigate the association of AKI with the length of ECMO need and mortality (35). The reported incidence of any stage AKI was 60%. Duration of ECMO support days and the need for renal replacement therapy were significantly higher in patients with AKI. The presence of AKI was associated with reduced survival to hospital discharge after controlling for multiple patients' characteristics and interventional support variables. In a separate analysis by the same group (36), the authors evaluated fluid overload as a modifiable risk of survival in critically ill children receiving ECMO. The median peak fluid overload was lower in patients who survived ECMO (27.2 vs. 44.4%) and those who survived until hospital discharge (24.8 vs. 43.3%).

### Impact of AKI on Pediatric Patients With Severe Sepsis

Sepsis is common in pediatric patients admitted to the pediatric intensive care unit. Severe sepsis occurs in around 8% of pediatric ICU patients (37).

Sepsis and shock were reported as the most frequent risk factors for AKI in critically ill children. A nationwide Taiwanese study that included more than 60,000 critically ill children found that 46.5% of the AKI cases were due to sepsis (38). A similar finding was reported in a study by Fitzgerald et al. in which AKI occurred in 42% of severe pediatric sepsis patients and severe AKI occurred in about 20% (8, 39).

The etiology of AKI in patients with severe sepsis seems to be multifactorial. This includes poor kidney perfusion secondary to septic shock, inflammatory mediators, kidney vasculature micro-thrombosis, kidney parenchymal ischemia and necrosis, and the use of nephrotoxic medications. In patients with warm shock, there is a decrease in the vascular resistance, with higher kidney perfusion, but lower GFR (glomerular filtration rate) secondary to changes in kidney microcirculation.

Fitzgerald et al. conducted one of the most important works regarding the impact of AKI on critically ill children. The authors did a *post hoc* analysis of the sepsis prevalence, outcomes, and therapies (SPROUT) study, which enrolled about 7,000 pediatric patients with severe sepsis from 128 pediatric intensive care units from over 26 countries (37). Twenty-one percent of critically ill children with severe sepsis, according to the 2005 international pediatric sepsis consensus criteria, developed stage II or III AKI. AKI carried an adjusted OR risk of 2.5 to have a composite outcome of death or new disability (39).

### Impact of AKI on Neonates

Premature and full-term neonates are unique in many health aspects. They are affected by the intrauterine environment, the stressful transition during delivery, and the postnatal complications.

AKI in neonates is multifactorial. Many risk factors were identified to complicate the ICU course of neonates. These risk factors can occur perinatally with early development of AKI or later during ICU admission. These risk factors include but are not limited to (1) prematurity, which is associated with incomplete nephrogenesis; (2) perinatal asphyxia and reperfusion injury following hypoxia (40, 41); and (3) the patency of the patent ductus arteriosus (PDA), which is associated with lower systemic vascular resistance and decreased kidney perfusion along with the use of NSAIDs to manage PDA (42). Other risk factors include low birth weight, congenital diaphragmatic hernia, bronchopulmonary dysplasia, and maternal nephrotoxic medications exposure like Angiotensin-Converting Enzyme (ACE) inhibitors (17). Other factors are associated with the late incidence of AKI such as sepsis, necrotizing enterocolitis with more than 50% of neonates with necrotizing colitis develop AKI (43), and the use of nephrotoxic medications in neonates like vancomycin, gentamicin, piperacillin-tazobactam, and amphotericin B. Some studies showed that more than 80% of premature babies receive at least one nephrotoxic medication (44, 45).

The prime attention of neonatal AKI was mainly highlighted in the last decade (46). The poor performance of serum creatinine and its fluctuated levels depending on the gestational and birth ages, especially in the first week of life in term babies and for a more extended period in premature babies, limited neonatal AKI research.

Several small, single-center studies in neonates with various primary morbidities such as congenital heart disease, hypoxic-ischemic injury, and very low birth weight infants suggest that AKI is common and that those with AKI have poor outcomes. A thorough AKI-core data were presented by the Assessment of Worldwide Acute Kidney Injury Epidemiology in Neonates (AWAKEN) study (47). AWAKEN is a comprehensive epidemiological study that was conducted to evaluate the impact of AKI in critically ill neonates. It included more than 2000 neonates admitted to 24 neonatal ICUs from Australia, Canada, India, and the United States. The study excluded infants admitted to NICU at 2 weeks of age or older, infants who underwent cardiovascular surgery repair of congenital heart disease within a week of life, infants diagnosed with lethal anomaly upon admission, and infants who died within 48 h of admission. The authors utilized the KDIGO definition of AKI with minor modifications to make the criteria applicable to neonates. The study showed that the overall incidence of AKI by meeting urine output or serum creatinine criteria was 29.9%. Neonates with gestational age < 29 weeks had the highest incidence rate of AKI (47.9%) (48). Infants who met the criteria for any stage AKI had a mortality rate of 9.7% compared to 1.4% without AKI. Within the AKI group, stage 3 AKI had higher mortality rates than stage 2 or stage 1. The impact of AKI on mortality was still significant after adjusting for multiple demographic characteristics, interventions, and comorbidities. Like other pediatric and adult literature, the authors reported longer hospital stay in neonates with AKI.

### Impact of AKI on Patients Post-stem Cell Transplantation

Hematopoietic stem cell transplantation (HSCT) is the transplantation of multipotent stem cells taken from bone marrow, umbilical cord, or peripheral blood to treat various diseases (e.g., leukemias, immunodeficiencies, and inborn errors of metabolism). HSCT is associated with numerous acute and long-term complications. The pre-HSCT conditioning, its intensity, preexisting comorbidities, chemotherapy exposure, the stem cell source, immunosuppression, and post-HSCT complications all make this population a unique population of critically ill children frequently admitted to pediatric ICUs. AKI is a well-known and common complication of HSCT in children. One of the first pediatric epidemiological studies of AKI in HSCT recipients using pRILFE criteria to define AKI was conducted in 2016 by Kizilbash et al. (49). They reported that 84% of patients developed AKI within 100 days post-HSCT, making AKI a prevalent HSCT complication. Previous pediatric studies have reported AKI in 21–42% of recipients within the first 100 days post-stem cell transplant; this wide range of incidence can be attributed to the difference in the AKI definitions used in different studies (50–52). In 2015, a systematic review study that included five observational studies showed that one-third of children post-HSCT developed AKI, with a median onset time of 4–6 weeks after transplantation (53).

There is a wide range of reported mortality rates in children with AKI post-HSCT. A published paper in 2003 about kidney function in children post-HSCT (54) showed that the mortality rate in the first 30 days post-HSCT was 19%, and the mortality rate in the patients who had AKI, defined as doubling in serum creatinine, was 55%. Lane et al. reported a mortality rate of 77% in children who developed severe AKI requiring dialysis after HSCT (55). In the systematic review study referenced earlier, the overall 100-day mortality for pediatric patients with AKI post-HSCT patients ranged from 10.5 to 12% (53). Kizilbash et al. (49) showed that the overall survival rate in pediatric patients post-HSCT was significantly lower among patients with AKI than patients without AKI (75 vs. 94%), and severe AKI was independently associated with increased mortality. There was a significant incremental increase in the mortality rate with increased AKI severity. Other studies showed that 5–10% of pediatric patients with severe AKI post-HSCT required RRT (50, 51, 54, 56), and the survival rate among them was only 42% (57).

Many risk factors for AKI have been reported in the literature, and these include unrelated donor, severe graft vs. host disease, severe infections, sepsis, veno-occlusive disease, the use of nephrotoxic medications (cyclosporine, amphotericin B, foscarnet, methotrexate, and calcineurin inhibitors), anemia, and total body irradiation (51, 52, 56, 58–60).

Post-HSCT AKI is associated with long-term complications. Kizilbash et al. (49) reported that 8% of children who developed AKI post HSCT and were alive at 1 year developed CKD. Other studies have reported CKD in 10% of children at 1-year post-HSCT (50–52, 56).

## The Impact of Volume Overload on Pediatric ICU Patients

Multiple factors make critically ill children vulnerable to volume overload. The systemic inflammation, capillary leak, and the arbitrary use of intravenous fluids to manage hypotension are among many factors that can contribute to volume overload. Regardless of meeting the AKI definition, the concept of volume overload has received extensive attention in the recent pediatric AKI and critical care literature. Despite the lack of a clear definition of volume overload and utilization of different formulas to identify it, multiple pediatric studies (61–64) found a good correlation between the percentile ratio of positive balance from ICU admission to weight upon admission (percent volume overload) and mortality rate. The percent volume overload can be calculated using different formulas. The following formula is one of the most commonly used formulas to calculate the percent volume overload:

[(total fluid intake (L) - total fluid output in liters (L))/(admission weight in kilograms) * 100] (64, 65).

By utilizing the above formula, Gillespie et al. (62) reported a death odds ratio (OR) of 3.02 for patients with >10% volume overload. Foland et al. (63) reported a death OR of 1.8 for each 10% volume overload increase and Hayes et al. reported an OR of 6.1 for volume overload of >20%.

However, such association between volume overload and mortality was not duplicated by Diaz et al. (66), who concluded that despite being common in children, volume overload in critically ill children was not an independent mortality risk factor after adjusting for other covariates. It is worth mentioning that AKI was an independent mortality risk factor in that study.

Selewski et al. evaluated the impact of volume overload in a specific ICU population. The group published a comprehensive 5-year multicenter evaluation of fluid overload and its association with patients' outcomes in children managed by ECMO (36). They found that fluid overload is common in children receiving ECMO, as more than 50% of patients had more than 10% fluid overload, and it was associated with prolonged ECMO duration and increased mortality independently when adjusted for other factors, including AKI.

Similarly, in patients with severe sepsis, Wong et al. found that cumulative fluid overload percent in the first 5 days of ICU admission was consistently and independently associated with increased mortality, fewer ventilator-free days, more inotrope utilization, and increased length of stay (67).

Two *post hoc* analyses from the AWAKEN study group evaluated the impact of volume overload in term and premature critically ill neonates (68, 69). The authors evaluated the percentile by calculating fluid balance during the first week of life using the formula: (daily weight – birth weight)/(birth weight × 100). The primary outcome was set for the need for mechanical ventilation on day 7 of life. The authors found that high positive fluid balance during the first week of life is independently associated with the primary outcome. However, the multivariate analysis did not show a solid association between positive volume balance and survival. The authors commented that the lack of statistical signal can be contributed to the low incidence of mortality in both cohorts of neonates.

A collective conclusion of the significance of volume overload in critically ill children regardless of the underlying etiology was highlighted in a systematic review and meta-analysis published in 2018 (70). In this work, the authors looked into more than 40, mainly retrospective studies, and found that the volume overload was associated with a 6% increase in adjusted risk of mortality. The study shed light on a major inconsistency among different studies in calculating and defining volume overload and its severity. Despite this, the authors concluded that their findings were robust and consistent in suggesting that fluid overload carried a greater risk for mortality and prolonged ventilator utilization.

Volume overload is common in critically ill children after cardiopulmonary resuscitation. Fluid resuscitation is a fundamental component of the metabolic phase of the three-phase pathophysiology model of cardiac arrest (71), and large volumes of intravenous fluids are often administered to increase the cardiac output and global oxygen delivery during this phase. However, the clinical course of these patients is commonly complicated with oliguric AKI and poor diuresis of the administered fluids. With the growing evidence of the deleterious impact of volume overload, conservative fluid management was proposed to decrease the risk of volume overload after resuscitation. A meta-analysis that included 49 studies concluded that conservative fluid strategy results in an increased number of ventilator-free days and a decreased length of ICU stay compared with a liberal strategy or standard care without observing a difference in mortality rates (72). Thus, when prescribing fluids during or after resuscitation, fluids should be used wisely. The conceptual model of “Four Phases of Intravenous Fluid Therapy” provides a pathophysiologic approach in managing the fluids around resuscitation time. These phases are rescue, optimization, stabilization, and de-escalation. More details about this model can be obtained from (73, 74).

## AKI and COVID-19 Critically Ill Children

According to the World Health Organization (WHO), since being declared as a global pandemic in March 2020 by the WHO, coronavirus disease 2019 (COVID-19) cases reached more than 115 million globally by the end of February 2021 as per the WHO COVID-19 dashboard. Pediatric COVID-19 cases account for around 1% of total COVID-19 cases reported by the Chinese center for disease control and prevention. Most children with COVID-19 had mild illnesses than adults, and only 0.6% of infected children have severe symptoms (75), with infants being affected more with severe illness (76, 77). While the early data in adults suggested that AKI was related to increased mortality risk, even after adjustment for age, sex, and comorbidities (78), the early data in children were not precise and probably conflicting. For example, one of the first studies describing AKI in COVID-19 children was published by Wang et al. (79), who reviewed 238 pediatric patients with confirmed COVID-19 from Wuhan Children's hospital; only three patients were sick and required ICU admission, and they developed AKI (incidence rate of 1.2%). The AKI in the three patients was part of the multisystem inflammatory syndrome and required supportive care with renal replacement therapy and plasma exchange. However, the data from Great Ormond Street Hospital in London (80) suggested a higher incidence of AKI than the Chinese study. AKI incidence was reported in 29% of 52 children with COVID-19 infection in the British report. AKI was again part of a multisystem inflammatory syndrome in most of the cases. None of the AKI cases required support with renal replacement therapy. To overcome this discrepancy, a multicenter cross-sectional study in SARS-CoV-2-infected critically ill children from six countries, but mainly from US centers, was conducted to evaluate the epidemiologic aspect of AKI in this cohort (81). The preliminary results of this study showed that among the 41 participating centers, only 26 centers reported COVID-19 children required ICU admission during the study period. Almost half (44%) of the enrolled critically ill children (a total of 106) developed AKI by KDIGO serum creatinine criteria. Despite the high prevalence, the AKI severity was not momentous as none of these patients needed dialysis despite the relatively high mortality rate among this cohort (6%). Diagnosis of shock and inotropic requirement at the time of admission were the main risk factors for AKI in univariate analysis. The authors concluded that the high prevalence of AKI in critically ill children would add more complexity to these children's medical care and recommended early identification of AKI to adjust the management accordingly.

## Conclusion

In this review, we depicted the impact of AKI in critically ill children in pediatric ICUs. The most recent literature consensus supports the conclusion that AKI is common in pediatric ICUs in general and in specific cohorts of critically ill children. AKI and its sequelae, such as volume overload, are considered consistent and independent risk factors for different outcomes such as mortality rates and ICU length of stay.

## Author Contributions

BL did the literature review and wrote the first draft. AK did literature review and finalized the submitted version of the manuscript. All authors contributed to the article and approved the submitted version.

## Conflict of Interest

The authors declare that the research was conducted in the absence of any commercial or financial relationships that could be construed as a potential conflict of interest.
